# Plant Food Delphinidin-3-Glucoside Significantly Inhibits Platelet Activation and Thrombosis: Novel Protective Roles against Cardiovascular Diseases

**DOI:** 10.1371/journal.pone.0037323

**Published:** 2012-05-18

**Authors:** Yan Yang, Zhenyin Shi, Adili Reheman, Joseph W. Jin, Conglei Li, Yiming Wang, Marc C. Andrews, Pingguo Chen, Guangheng Zhu, Wenhua Ling, Heyu Ni

**Affiliations:** 1 Canadian Blood Services, Toronto, Ontario, Canada; 2 Department of Nutrition, School of Public Health, Sun Yat-sen University (Northern Campus), Guangzhou, People's Republic of China; 3 Toronto Platelet Immunobiology Group, Department of Laboratory Medicine, and Keenan Research Centre in the Li Ka Shing Knowledge Institute of St. Michael's Hospital, Toronto, Ontario, Canada; 4 Department of Laboratory Medicine and Pathobiology, University of Toronto, Toronto, Ontario, Canada; 5 Department of Physiology, University of Toronto, Toronto, Ontario, Canada; 6 Department of Medicine, University of Toronto, Toronto, Ontario, Canada; Heart Center Munich, Germany

## Abstract

Delphinidin-3-glucoside (Dp-3-g) is one of the predominant bioactive compounds of anthocyanins in many plant foods. Although several anthocyanin compounds have been reported to be protective against cardiovascular diseases (CVDs), the direct effect of anthocyanins on platelets, the key players in atherothrombosis, has not been studied. The roles of Dp-3-g in platelet function are completely unknown. The present study investigated the effects of Dp-3-g on platelet activation and several thrombosis models *in vitro* and *in vivo*. We found that Dp-3-g significantly inhibited human and murine platelet aggregation in both platelet-rich plasma and purified platelets. It also markedly reduced thrombus growth in human and murine blood in perfusion chambers at both low and high shear rates. Using intravital microscopy, we observed that Dp-3-g decreased platelet deposition, destabilized thrombi, and prolonged the time required for vessel occlusion. Dp-3-g also significantly inhibited thrombus growth in a carotid artery thrombosis model. To elucidate the mechanisms, we examined platelet activation markers via flow cytometry and found that Dp-3-g significantly inhibited the expression of P-selectin, CD63, CD40L, which reflect platelet α- and δ-granule release, and cytosol protein secretion, respectively. We further demonstrated that Dp-3-g downregulated the expression of active integrin αIIbβ3 on platelets, and attenuated fibrinogen binding to platelets following agonist treatment, without interfering with the direct interaction between fibrinogen and integrin αIIbβ3. We found that Dp-3-g reduced phosphorylation of adenosine monophosphate-activated protein kinase, which may contribute to the observed inhibitory effects on platelet activation. Thus, Dp-3-g significantly inhibits platelet activation and attenuates thrombus growth at both arterial and venous shear stresses, which likely contributes to its protective roles against thrombosis and CVDs.

## Introduction

Platelets are small anucleate cells in the blood that can sense environmental changes, including the alteration of nutrition products of food in the blood circulation [1]–[4]. Platelets are versatile cells and play important roles in hemostasis/thrombosis, inflammation, and atherosclerosis [5], [6]. Platelet adhesion and subsequent aggregation at the site of vascular injury are key events required for hemostasis. However, excessive platelet accumulation and thrombus formation may result in thrombotic diseases such as myocardial infarction or stroke, the two leading causes of morbidity and mortality worldwide. Many cardiovascular diseases (CVDs), including the initiation of atherosclerosis, are linked to platelet hyperactivity, the abnormal, excessive activation of platelets, which is considered an independent risk factor for CVDs [7], [8].

At the site of vascular injury, particularly at high shear stress, such as rupture of atherosclerotic lesions in coronary arteries, the binding of the platelet glycoprotein (GP)Ib complex to von Willebrand factor (VWF) on the injured vessel wall initiates platelet tethering and subsequent adhesion. Platelet aggregation is then mediated by interaction between platelet αIIbβ3 integrin and fibrinogen (Fg), although Fg-independent platelet aggregation, as we demonstrated, can also occur [9]–[11]. Platelet activation, induced by platelet agonists such as adenosine diphosphate (ADP), collagen, and thrombin at the site of injury, is an important process for platelet adhesion and aggregation. Following activation, platelet granule and cytosol proteins, such as P-selectin (CD62P), CD63 and CD40L, may translocate to the cell surface. Platelet activation also results in the conversion of αIIbβ3 integrin to an active conformation, allowing it to bind ligands including Fg and other proteins [9]–[11]. The translocation and conformational changes of these proteins after platelet activation facilitates the interaction of platelets with their environment (e.g. extracellular matrix proteins on the vessel wall and αIIbβ3 integrin ligands in the blood during platelet adhesion and aggregation), which are important processes for hemostasis and thrombosis, as well as for inflammation and atherosclerosis. Therefore, downregulating platelet activation may be a feasible approach for preventing and treating CVDs resulting from platelet hyperactivation.

Mounting epidemiological information suggests that dietary intake of plant foods rich in phytochemicals is negatively associated with CVDs [12]. The polyphenols are a well-studied group of phytochemicals, among which the anthocyanins have been shown to impart significant beneficial properties [13], [14]. Anthocyanins are abundant in various fruits and vegetables and their related products, including grapes, berries, red cabbage and red wine. Our previous studies, in addition to those of other research groups, have demonstrated that intake of anthocyanin-rich beverages or pure anthocyanins inhibits atherosclerosis through anti-oxidative and anti-inflammatory properties [15], [16], as well as by improving lipid profiles and endothelium-dependent vasodilatation [15], [17]–[19]. Furthermore, some studies have shown that dietary supplementation with anthocyanins imparts anti-thrombotic effects by modulating platelet aggregation and function in human whole blood and platelet-rich plasma (PRP) [20]. Our recent studies have also shown that anthocyanin extract from black rice facilitates the maintenance of optimal platelet function in dyslipidemic rats consuming high fat diets [3]. In contrast, some studies suggested that steady consumption of anthocyanin supplements did not significantly affect platelet aggregation [21], [22]; therefore, the conclusions drawn from human studies are contradictory and further investigation of the effects of anthocyanins on platelet function are required.

In addition to differences in methodologies, the seemingly contradictory results from studies evaluating the effects of anthocyanins on platelet function may be because the majority of studies have focused on polyphenolic-rich foods or beverages, such as grape juice [23] and red wine [24], which contain multiple compounds and different concentrations or subclasses of anthocyanins. Other important factors to consider are that in humans, the rate of absorption and elimination of, as well as the relative bioavailability of, anthocyanins present in different foods is unclear. Therefore, it is important to examine the direct effects of pure anthocyanins on platelet function in order to elucidate their mechanisms of action *in vitro* and *in vivo*. To date, the anti-platelet activity of four purified anthocyanins (delphinidin-3-rutinoside, cyanidin-3-glucoside, cyanidin-3-rutinoside, and malvidin-3-glucoside) have been assayed in human whole blood and PRP [20]. However, to the best of our knowledge, the direct role of purified anthocyanins on platelet function and their effects on thrombus formation under flow conditions has not been studied.

Delphinidin-3-glucoside (Dp-3-g), one of the predominantly bioactive compounds of anthocyanin preparations, is a natural colorant found in bilberries and other fruits and flowers [25]. Some studies have shown that it has beneficial effects on human low-density lipoprotein cholesterol oxidation [26]; however, differing from other anthocyanin compounds, its roles in platelet function (including in PRP) and thrombosis are completely unknown. In the present study, we determined whether Dp-3-g could alter platelet aggregation in both PRP and purified gel-filtered platelets, as well as in several thrombosis models *in vitro* and *in vivo*. We found that Dp-3-g significantly inhibits platelet aggregation, and attenuates thrombus growth under both arterial and venous shear stresses. We demonstrated that Dp-3-g inhibited platelet activation and attenuated platelet adenosine monophosphate-activated protein kinase (AMPK) phosphorylation, which may explain its protective role against thrombosis and other CVDs.

## Materials and Methods

### Ethics Statement

For research involving human participants, written informed consent was obtained in accordance with the Declaration of Helsinki and studies were approved by the St. Michael's Hospital Research Ethics Board. All animal procedures were approved by the Animal Care Committee at St. Michael's Hospital (protocol numbers 213 and 181). Mice were housed in the research vivarium at St. Michael's Hospital.

### Reagents

Dp-3-g was purchased from Polyphenol AS (Norway). Thrombin receptor activating peptide (TRAP; AYPGKF-NH_2_) was purchased from Peptides International (Louisville, MO, USA). Thrombin and ADP were purchased from Sigma-Aldrich (Oakville, ON, Canada). Type-I collagen fibrils (equine collagen Horm) was purchased from Nycomed (Roskilde, Denmark). DiOC6 dye was purchased from Invitrogen (Burlington, ON, Canada). PE-conjugated mouse anti-human PAC-1 antibody, FITC-conjugated mouse anti-human CD63 antibody, FITC-conjugated mouse anti-human CD40L antibody, FITC-conjugated mouse anti-human CD62P antibody, PE-conjugated mouse anti-mouse CD62P antibody, FITC-conjugated mouse IgG1κ Isotype control, PE-conjugated anti-mouse IgG antibody, FITC-conjugated mouse anti-human IgG1κ Isotype control and PE-conjugated anti-human IgG1κ Isotype control were purchased from BD Biosciences (Mississauga, ON, Canada). PE-conjugated goat anti-mouse JON/A antibody was purchased from Emfret Analytics (Eibelstadt, Germany). Rabbit antibodies against phosphorylated AMPK (pAMPK, Thr172) and AMPK were purchased from Cell Signaling Technology (Danvers, MA, USA). Fg from human plasma, Alexa Fluor 488 conjugate and Calcein AM was purchased from Invitrogen (Burlington, ON, Canada).

### Mice

C57BL/6J mice were purchased from Charles River Laboratories International (Wilmington, MA).

### Murine platelet and plasma preparation

Mice (6–8 weeks old) were anesthetized and bled from the retro-orbital plexus with the use of heparin-coated glass capillary tubes. Blood was collected into tubes containing either 3% ACD (1/9 vol/vol) or 25 U/mL heparin. PRP was obtained by centrifugation at 300 g for 7 min. Platelet-poor plasma (PPP) was prepared by centrifugation at 1500 g for 20 min. The PPP was further centrifuged at 10,000 g for 5 min to remove any remaining cells. Gel-filtered platelets were prepared as previously described [27], [28]. Briefly, platelets were isolated from citrated PRP using a Sepharose 2B column in PIPES buffer (5 mM PIPES, 1.37 mM NaCl, 4 mM KCl, 0.1% (wt/vol) glucose, pH 7.0).

### Human blood preparation

Blood was obtained from healthy human subjects who had not taken any anti-platelet medication in the prior two week period. Blood was obtained by venupuncture into Li-Heparin Vacutainers and was allowed to rest at 37°C for 10 min prior to preparation of PRP. Whole anticoagulated blood was spun at 300 g for 7 min. The PRP was transferred to a fresh tube and stored at 37°C until use. Gel-filtered platelets were prepared using the same method as for preparation of murine gel-filtered platelets.

### Platelet Aggregation

Aggregation of PRP and gel-filtered platelets were performed at 37°C with a sample stir speed of 1000 rpm using a computerized aggregometer (Chrono-Log Corp, Havertown, PA) as we previously described [10], [11]. PRP and gel-filtered platelets were pre-incubated with the indicated doses of anthocyanin or control buffer for 40 min at 37°C. For PRP, the baseline was adjusted with PPP, and for gel-filtered platelets, equal amounts of platelets were mixed with PIPES (final concentration ∼2.5×10^8^ platelets/mL). A total of 250 µL of each sample was added to an aggregation cuvette and incubated for 2 min. Aggregation was induced by 5 µM ADP, 100 µM TRAP, 0.1 U/mL thrombin or 2 µg/mL collagen in the presence of 1 mM Ca^2+^. Aggregation was recorded for 8 min, and data were expressed as a percentage of maximum aggregation of the control without pre-incubation with Dp-3-g.

### Perfusion flow assays

The *ex vivo* perfusion chamber thrombosis model was performed at low (600 s^−1^) and high (1800 s^−1^) shear rates, as we previously described [10]. Briefly, rectangular (0.1×1 mm) glass capillary microslides were coated with 100 µg/mL type-I collagen fibrils overnight at 4°C. Where indicated, whole blood was pre-incubated with different levels of Dp-3-g or control buffer alone for 40 min at 37°C prior to perfusion, followed by washout with phosphate buffered saline (PBS). Platelet adhesion, aggregation, and thrombus formation were recorded in real-time over the course of perfusion under bright field with a Zeiss Axiovert 135 inverted microscope and computer (IBM IntelliStation Z Pro) using the Slidebook program (Intelligent Imaging Innovations). Surface coverage and thrombus size were calculated from the light microscope images using ImageJ software.

### Intravital microscopy thrombosis models

The process of thrombus formation in arterioles was monitored in 3–4 week old mice, as we previously described [9]–[11], [27], [29]. Briefly, blood was collected into ACD (1∶10) from the experimental mice. PRP was prepared by centrifugation. Platelets were separated from plasma by gel-filtration, labeled with 1 mg/mL of Calcein AM at room temperature for 20 min, and then injected into the experimental mouse (1.25×10^6^ platelets/g) via the tail vein. Mice were then anesthetized and the mesentery was externalized. In each mouse, a single mesenteric arteriole of 100–120 µm diameter was chosen and injury was induced by topical application of 30 µL of 250 mM FeCl_3_. Control buffer or Dp-3-g was injected via the tail vein 40 min before FeCl_3_ application. Images of thrombus formation and dissolution were visualized with a fluorescence microscope (Zeiss Axiovert 135; Zeiss Oberkochen) and compared between groups based on the time to complete vessel occlusion, as previously described [27], [29].

### Carotid artery thrombosis model

C57BL/6 mice (6-weeks old) were injected with 50 µM Dp-3-g or control buffer via the tail vein. Following anesthetization, the right carotid artery was dissected and held with a miniature Doppler flow probe (TS420 transit-time perivascular flowmeter, Transonic Systems Inc.). Carotid artery injury was induced with a strip of Whatman filter paper saturated with 10% ferric chloride [30]. The blood flow was monitored until complete vessel occlusion was observed.

### Bleeding time assay

C57BL/6 mice (6–8 weeks old) were injected with either control buffer or Dp-3-g intravenously via the tail vein 40 min before the bleeding time was assessed. Mice were anesthetized with 2.5% tribromoethanol (0.015 mL/g) and maintained at 37°C on a heating pad during the experiment. The tip of the tail (5 mm) was cut off with a sharp scalpel, and the tail was immediately placed into warm saline at 37°C. The bleeding time was the period from the moment blood began emerging from the cut until the moment bleeding ceased.

### Detection of P-selectin expression on human and mouse platelets

Human and mouse PRP and gel-filtered platelets were incubated with different concentrations of Dp-3-g or control buffer for 40 min at 37°C. Aliquots of sample (100 µL) were transferred to tubes containing saturating concentrations of the following fluorescently-labeled monoclonal antibodies: PE-conjugated anti-human CD62P antibody, FITC-conjugated anti-mouse CD62P antibody, PE/FITC-conjugated anti-mouse/human IgG1κ (isotope controls), and incubated for 15 min at room temperature. PRP or gel-filtered platelets were activated with either 200 µM ADP, 250 µM TRAP, 10 µg/mL collagen or 1 U/mL thombin, for 5 min at room temperature. PBS (0.5 mL) was added to each sample immediately before acquisition. All samples were analyzed via a calibrated FACSCalibur flow cytometer (BD Biosciences). Ten thousand events per sample were acquired; light scatter and fluorescence channels were set at a logarithmic gain, the platelet population was analyzed for mean fluorescence intensity (MFI). For each experiment, all samples for comparison were acquired at the same settings.

### Detection of CD63 and CD40L expression on human platelets

Human gel-filtered platelets were incubated with different concentrations of Dp-3-g or control buffer for 40 min at 37°C. To label CD63 and CD40L, platelets were incubated with FITC-conjugated mouse anti-human CD63 or anti-human CD40L for 15 min at room temperature. Gel-filtered platelets were activated with 1 U/mL thrombin, 250 µM TRAP or 10 µg/mL collagen in the presence of 1 mM Ca^2+^ for 5 min. The platelets were fixed with 1% paraformaldehyde before being analyzed via flow cytometry.

### Detection of activated integrin αIIbβ3 expression on human and mouse platelets

The expression of activated integrin αIIbβ3 on human and murine platelets, measured by PAC-1 and JON/A antibody, respectively, were tested as we previously described [31]. PRP or gel-filtered platelets were incubated in polystyrene tubes for 40 min at 37°C with different concentrations of Dp-3-g or control buffer. Aliquots of sample (100 µL) were transferred to tubes containing saturating concentrations of the following fluorescently-labeled monoclonal antibodies: FITC-conjugated anti-human PAC-1 antibody, FITC-conjugated anti-human IgG1κ (isotype control), PE-conjugated anti-mouse JON/A antibody or PE-conjugated mouse IgG1κ (isotype control), and incubated for 15 min at room temperature. Samples in the presence or absence of the agonists 200 µM ADP, 250 µM TRAP, or 10 µg/mL collagen were incubated for 5 min at room temperature. The samples were then fixed in filtered 1% paraformaldehde (pH 7.2) and stored in the dark at 4°C. All samples were analyzed via flow cytometry.

### Detection of platelet-bound fibrinogen on human and mouse platelets

Resting gel-filtered platelets were incubated with different concentrations of Dp-3-g or control buffer for 40 min at 37°C, and were then incubated with FITC-conjugated Fg for 15 min at room temperature. Platelet activation was induced by 200 µM ADP, 250 µM TRAP, 10 µg/mL collagen or 1 U/mL thrombin for 5 min at room temperature. The samples were then fixed in filtered 1% paraformaldehyde (pH 7.2) and stored in the dark at 4°C. All samples were analyzed via flow cytometry.

### Detection of threonine phosphorylation of AMPK in human and mouse platelets

Gel-filtered platelets were pre-incubated with the indicated doses of Dp-3-g or control buffer for 40 min at 37°C. Platelets were activated with 25 µg/mL collagen for 90 s at room temperature. Platelets were solubilized in Triton X-100 lysis buffer, and 20 µg of soluble proteins were separated by sodium dodecyl sulfate–polyacrylamide gel electrophoresis (SDS-PAGE). Western blotting was performed as previously described [32]. Membranes were blocked with 5% (w/v) skim milk powder in Tris-buffered saline-Tween (TBS-T; 20 mM Tris, 137 mM NaCl, 0.1% (v/v) Tween 20, pH 7.6). Primary antibody that specifically recognizes phosphorylated AMPK (pAMPK) residue Thr172 and horseradish peroxide (HRP)-conjugated secondary antibody (anti-rabbit IgG-HRP) were diluted 1∶1000 and 1∶2000, respectively, in TBS-T containing 5% (w/v) bovine serum albumin (BSA). Blots were developed with ECL detection reagents (Thermo Scientific) according to the manufacturer's instructions. Following detection, the same membranes were stripped with stripping buffer and re-blotted with a primary antibody that recognizes total AMPK.

### Statistical analysis

Data from each treatment or control group were analyzed by one-way ANOVA coupled with the Student-Newman-Keuls multiple comparison test using the SPSS 16.0 statistical package. Differences were considered significant if *P*<0.05.

## Results

### Dp-3-g inhibited platelet aggregation following stimulation of platelets with various agonists

**Figure 1:**
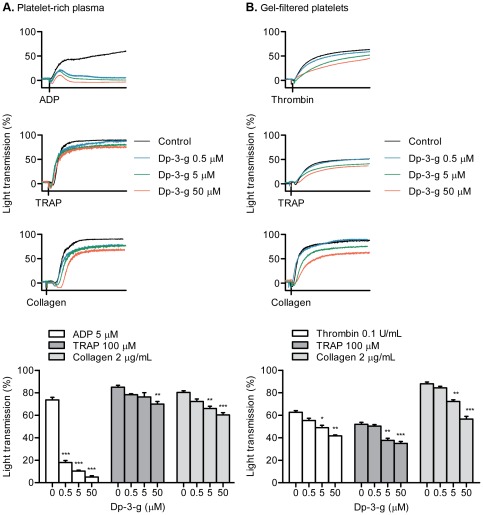
Effects of Dp-3-g on human platelet aggregation. Human PRP and gel-filtered platelets were pre-incubated with control buffer (black), 0.5 µM Dp-3-g (blue), 5 µM (green) or 50 µM Dp-3-g (red) for 40 min at 37°C. Platelet aggregation with human PRP or gel-filtered platelets was performed at 37°C with a stir speed of 1000 rpm using an aggregometer. A) Human PRP. B) Human gel-filtered platelets. Values are mean ± SD, n = 3 per group. * *P*<0.05, ** *P*<0.01 and *** *P*<0.001, as compared to control buffer.

The effects of Dp-3-g on platelet function have not been previously explored. We found that Dp-3-g significantly inhibited platelet aggregation following stimulation with ADP, collagen or TRAP in both human PRP (**Figure 1A**) and mouse PRP (**Figure S1A**) in a dose-dependent manner. Dp-3-g markedly inhibited ADP-induced human platelet aggregation at doses as low as 0.5 µM, although significant inhibition of platelet aggregation induced by the strong agonists collagen and TRAP was observed at higher doses. To distinguish whether this inhibition was directly due to the effect of Dp-3-g on platelets but not due to indirect effects following possible interactions with other blood components, human and mouse platelets were isolated via gel-filtration. Dp-3-g significantly inhibited aggregation of purified human platelets induced by collagen, thrombin or TRAP (*P*<0.01) in a dose-dependent manner (**Figure 1B**). Dp-3-g also significantly inhibited aggregation of purified mouse platelets induced by these agonists. Significant inhibition of collagen- and TRAP-induced platelet aggregation was observed at Dp-3-g doses as low as 0.5 µM (**Figure S1B**).

### Dp-3-g inhibited human and mouse thrombus formation in *ex vivo* flow chambers

**Figure 2:**
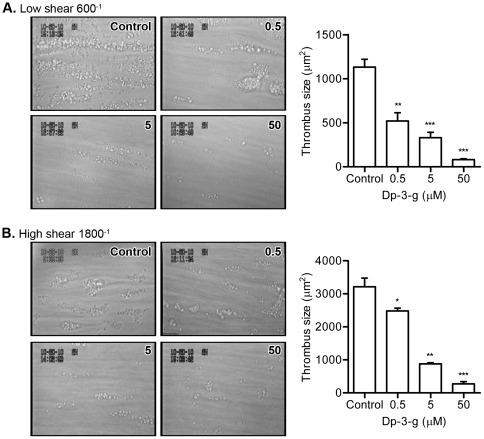
Effects of Dp-3-g on human thrombus formation under flow. *Ex vivo* thrombus formation was monitored on type-I collagen at 600 s^−1^ or 1800 s^−1^ using human whole blood with different concentration of Dp-3-g or control buffer. A) Low shear rate of 600 s^−1^. B) High shear rate of 1800 s^−1^. Values are mean ± SEM, n = 5 per group.* *P*<0.05, ** *P*<0.01 and *** *P*<0.001, as compared to control buffer.

To evaluate the effects of Dp-3-g on platelet thrombus formation under flow conditions, we performed perfusion experiments using glass coverslips coated with type I collagen fibrils. Consistent with the data from *in vitro* platelet aggregation, we observed that Dp-3-g significantly inhibited human thrombus formation at both low (600 s^−1^) (**Figure 2A**) and high shear rates (1800 s^−1^) (**Figure 2B**) in a dose-dependent manner. Significant inhibition was observed at doses as low as 0.5 µM under both the low (*P*<0.01) and high shear rates (*P*<0.05). Marked inhibition of mouse thrombus growth was also observed following the addition of different concentrations of Dp-3-g to heparin-anticoagulated murine blood (**Figure S2A–S2B**).

### Dp-3-g inhibited murine thrombus growth *in vivo*

**Figure 3:**
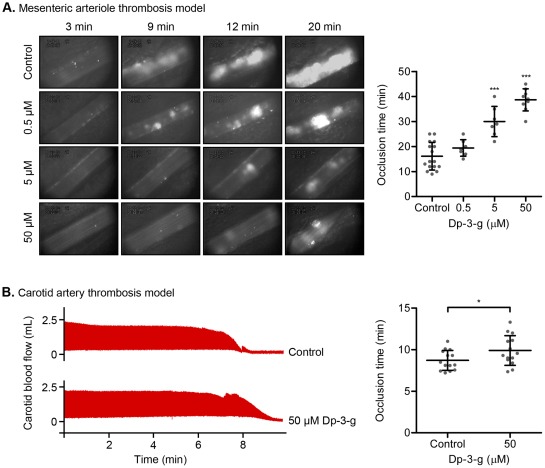
Effects of Dp-3-g on FeCl_3_-induced thrombosis *in vivo*. A) Thrombus formation was initiated by topical application of FeCl_3_ on mesenteric arterioles in C57BL/6 male mice, which were injected with fluorescently-labeled platelets and different concentration of Dp-3-g or control buffer. Thrombus formation was compared between groups based on the time to complete vessel occlusion. Values are mean ± SD, n = 6–10 per group. *** *P*<0.001, as compared to control buffer. B) C57BL/6 mice were injected with 50 µM Dp-3-g or control buffer. Blood flow in the carotid artery following FeCl_3_-induced injury was monitored until complete vessel occlusion was observed. Values are mean ± SD, n = 14 per group. * *P*<0.05.

To test the effects of anthocyanin on thrombus formation *in vivo*, we first used FeCl_3_-induced mesenteric arteriole injury in an intravital microscopy thrombosis model. We found that Dp-3-g decreased platelet deposition and prolonged the time required for thrombus formation and vessel occlusion (**Figure 3A**). Compared to the control group, thrombus growth was reduced at a dose of 0.5 µM and vessel occlusion was significantly delayed in the 5 µM and 50 µM Dp-3-g treatment groups (*P*<0.01).

We further examined this effect on large vessels using a carotid artery thrombosis model [30]. We found that vessel occlusion time was significantly delayed in the 50 µM Dp-3-g treatment group (*P*<0.05) compared to the control group (**Figure 3B**).

### Dp-3-g did not significantly affect bleeding time in mice

**Figure 4:**
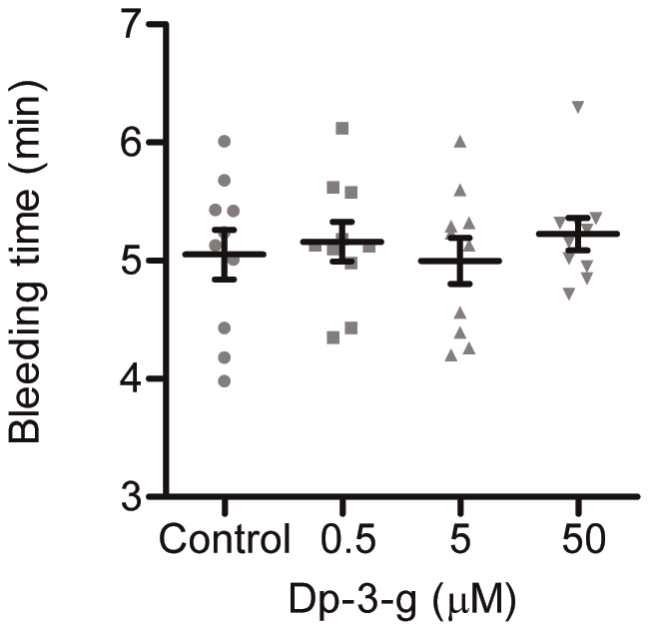
Effects of Dp-3-g on bleeding times in mice. Tail-vein bleeding times were examined in C57BL/6 mice. Either PBS (control) or different concentrations of Dp-3-g were administered via the tail vein 40 min before the bleeding time was determined. Values are mean ± SD, n = 8–10 per group. No significant differences in bleeding times were observed between treated and untreated mice.

To investigate the effect of Dp-3-g on normal hemostasis, bleeding times were measured 40 minutes after Dp-3-g or control buffer was injected intravenously via the mouse tail vein. No significant difference in tail-bleeding times was observed in wild-type mice treated with different doses of Dp-3-g versus control buffer (**Figure 4**). This is consistent with an earlier observation that thrombosis is more sensitive than hemostasis in response to platelet inhibitors [33].

### Dp-3-g inhibited P-selectin expression on human and murine platelets after agonist stimulation

**Figure 5:**
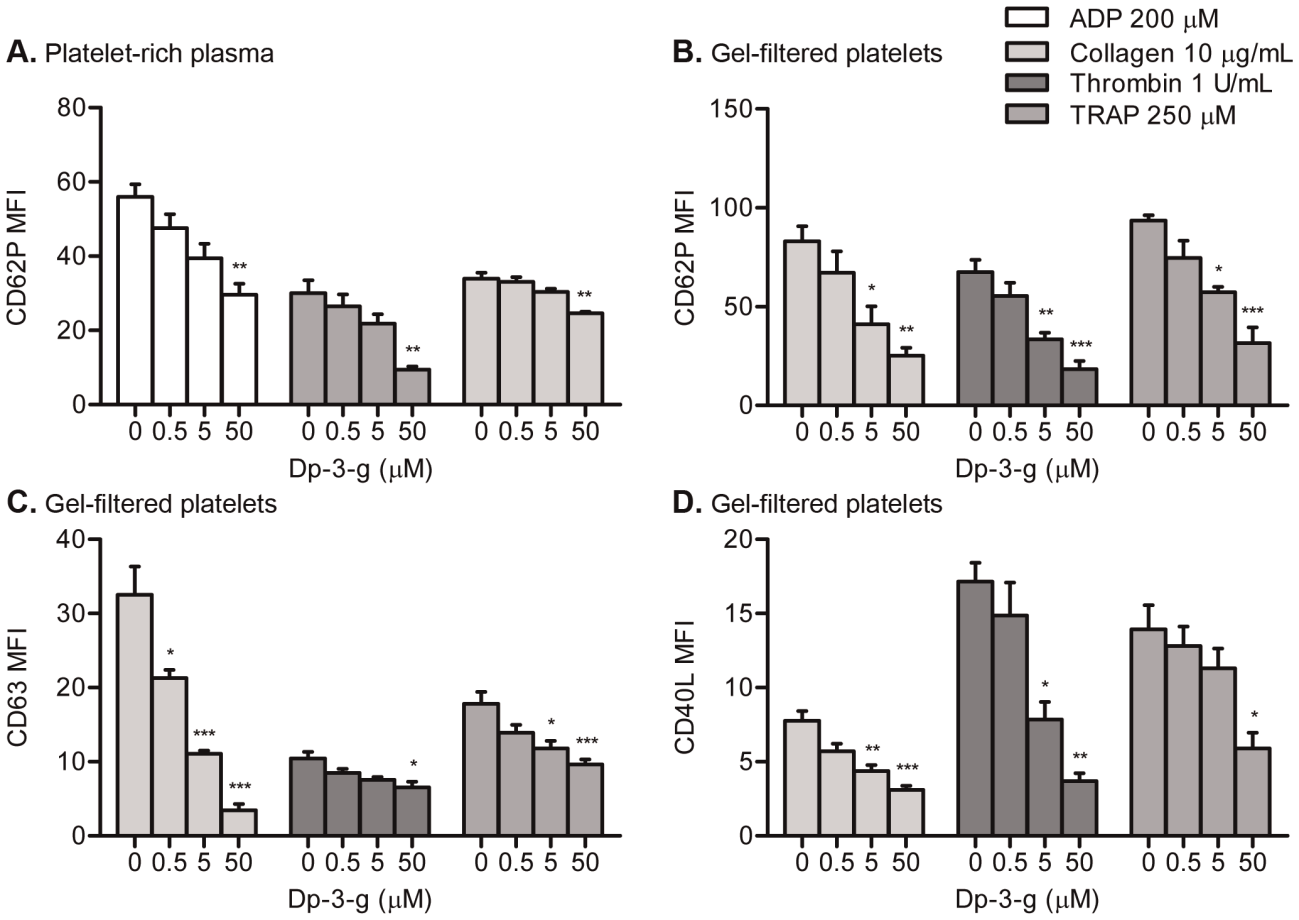
Effects of Dp-3-g on human platelet activation. Human PRP or gel-filtered platelets were incubated with control buffer, 0.5 µM Dp-3-g, 5 µM Dp-3-g or 50 µM Dp-3-g for 40 min at 37°C. Platelet activation markers were analyzed via flow cytometry after stimulation by ADP, collagen, TRAP or thrombin. A) P-selectin expression on human PRP. B) P-selectin expression on human gel-filtered platelets. C) CD63 expression on human gel-filtered platelets. D) CD40L expression on human gel-filtered platelets. Values are mean ± SEM, n = 3 per group. * *P*<0.05, ** *P*<0.01 and *** *P*<0.001, as compared to control buffer.

To investigate the effects of anthocyanin Dp-3-g on platelet activation, P-selectin expression on human and murine platelets was examined via flow cytometry. We found that Dp-3-g inhibited platelet P-selectin expression after stimulation with high doses of ADP, collagen or TRAP (**Figure 5A**). Significant inhibition was observed with 50 µM Dp-3-g (*P*<0.05) for human PRP. The inhibitory effect was also observed at 5 µM and 50 µM Dp-3-g (*P*<0.05) for mouse PRP (**Figure S3A**). Thus, Dp-3-g inhibited platelet α-granule release in PRP.

It is currently unknown whether the anthocyanin Dp-3-g can directly affect platelet activation or act on other molecules in blood plasma that indirectly affect the platelet activation process. We therefore examined P-selectin expression on purified gel-filtered platelets. Significantly less P-selectin expression was observed following stimulation by collagen, thrombin or TRAP at 5 µM and 50 µM Dp-3-g (*P*<0.05). The MFI decreased approximately 50% and 70% compared with controls (**Figure 5B**). The weak agonist ADP was not used for these experiments since it is usually unable to induce granule release in gel-filtered platelets. Consistent with human gel-filtered platelets, a significant decrease in P-selectin on mouse purified platelets was also observed (**Figure S3B**). These data clearly demonstrate that Dp-3-g can directly inhibit platelet P-selectin expression in a dose-dependent manner.

### Dp-3-g significantly inhibited CD63 and CD40L expression on human platelets

Recent research has suggested that platelets can sense different signals during activation and selectively release their granules [34], [35]. We therefore examined the δ-granule secretion marker, CD63, and a platelet cytoplasmic inflammatory factor, CD40L. Since expression of these markers is relatively low, we used strong agonists to induce platelet activation in gel-filtered platelets. Dp-3-g significantly decreased both CD63 and CD40L expression on human gel-filtered platelets following stimulation with various agonists (*P*<0.05) (**Figure 5C–5D**), and was able to inhibit CD63 expression at concentrations as low as 0.5 µM. Thus, in addition to its inhibitory effect on platelet α-granules, Dp-3-g can also inhibit platelet δ-granule release, as well as cytosol protein secretion, in a dose-dependent manner.

### Dp-3-g significantly inhibited expression of active integrin αIIbβ3 on human and mouse platelets

**Figure 6:**
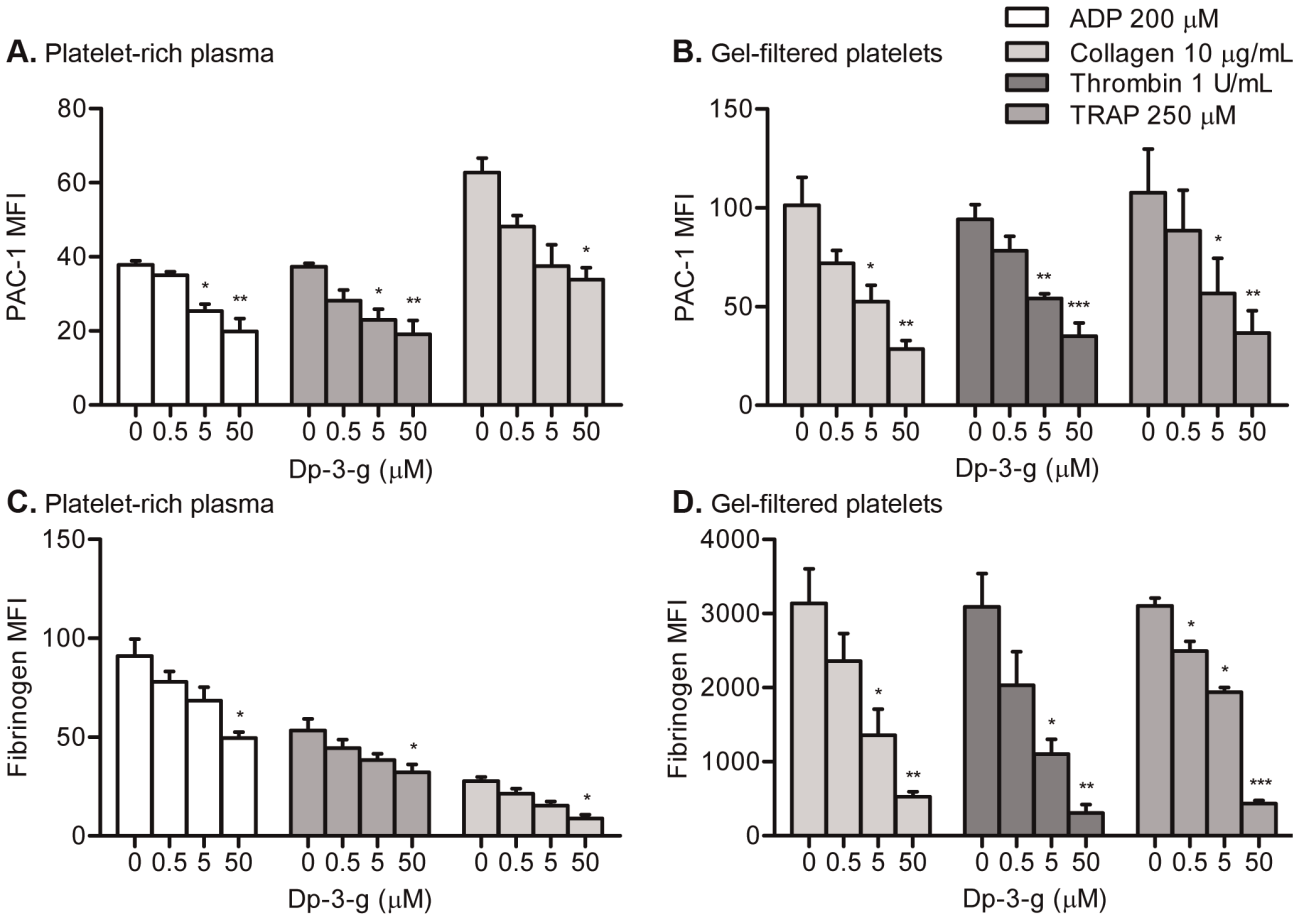
Effects of Dp-3-g on human platelet αIIbβ3 activation and fibrinogen binding. Human PRP or gel-filtered platelets were incubated with control buffer, 0.5 µM Dp-3-g, 5 µM Dp-3-g or 50 µM Dp-3-g for 40 min at 37°C. Platelet activation markers were analyzed via flow cytometry after stimulation by ADP, collagen, TRAP or thrombin. A) Activated integrin αIIbβ3 expression on platelets in human PRP. B) Activated integrin αIIbβ3 expression on human gel-filtered platelets. C) Platelet-bound fibrinogen in human PRP. D) Platelet-bound fibrinogen on human gel-filtered platelets. Values are mean ± SEM, n = 3 per group. * *P*<0.05, ** *P*<0.01 and *** *P*<0.001, as compared to control buffer.

Integrin αIIbβ3, an abundant platelet membrane protein, is required for platelet aggregation [11], [36]. During platelet activation, αIIbβ3 switches from an inactive to an active conformation, which reveals the epitope for the monoclonal antibodies, PAC-1 and JON/A for human and murine αIIbβ3, respectively. We found that Dp-3-g significantly inhibited platelet PAC-1 binding in both human PRP and gel-filtered platelets following platelet stimulation with ADP, collagen, TRAP or thrombin (*P*<0.05) (**Figure 6A–6B**). It also inhibited murine platelet JON/A binding in mouse PRP and gel-filtered platelets (*P*<0.05) (**Figure S3C–S3D**).

### Dp-3-g significantly inhibited binding of fibrinogen to human and mouse platelets

Fg is a major ligand of integrin αIIbβ3 and the engagement of Fg with activated αIIbβ3 plays a key role in platelet aggregation and thrombus formation. We found that Dp-3-g significantly inhibited Fg binding to both human PRP and gel-filtered platelets after stimulation by ADP, collagen, TRAP or thrombin (*P*<0.05) (**Figure 6C–6D**). Similar effects were also observed in murine PRP and gel-filtered platelets (*P*<0.05) (**Figure S3E–S3F**). This inhibitory effect is unlikely due to a direct interaction between Dp-3-g and αIIbβ3 (data not shown) as no competition was observed between Fg and purified αIIbβ3 integrin-coated wells via ELISA or αIIbβ3 integrin-coated latex beads via flow cytometry.

### Dp-3-g significantly inhibited AMPK phosphorylation in human and mouse platelets

**Figure 7:**
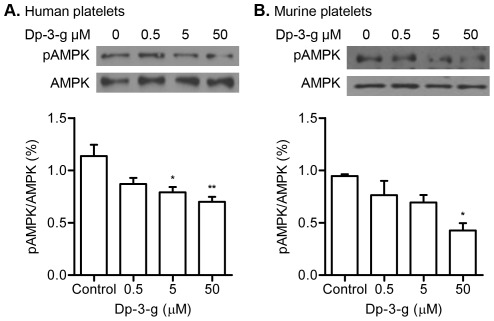
Effects of Dp-3-g on human and murine platelet phosphorylation of threonine residues of AMPK. Platelets activated with collagen in the presence of Dp-3-g were lysed and proteins were separated by SDS–PAGE and immunoblotted to detect phospho-threonine residues. A) Western blot analysis of AMPK phosphorylation in human platelets and the associated percent-change observed when compared with controls. B) Western blot analysis of AMPK phosphorylation in murine platelets and the associated percent-change observed when compared with controls. Values are mean ± SD, n = 3 per group. * *P*<0.05 and ** *P*<0.01, as compared to control buffer.

AMPK is a key sensor of cellular energy status [37], and platelet activation is an energy-consuming process. Our group, as well as other laboratories, recently demonstrated that anthocyanins affect AMPK phosphorylation in cells of several distinctive tissues [32], [38], [39], but their effect on platelet AMPK has not been explored. To determine whether Dp-3-g affects the phosphorylation of AMPK, which has recently been implicated in platelet signalling [40], platelet pAMPK levels were examined following collagen activation. Compared to the control group, pAMPK levels in human platelets were reduced in a dose-dependent manner and significant reduction was observed in the presence of 5 and 50 µM Dp-3-g (*P*<0.05 and *P*<0.01, respectively) (**Figure 7A** **)**. A similar dose-dependent reduction in pAMPK was also observed in collagen-activated murine platelets (*P*<0.05) (**Figure 7B** **)**.

## Discussion

The present study demonstrates that Dp-3-g, one of the predominantly bioactive compounds of anthocyanins, inhibits platelet activation and aggregation, as well as attenuates thrombosis *in vitro* and *in vivo*. To the best of our knowledge, this is the first study that clearly demonstrates that an anthocyanin can directly inhibit platelet function, in addition to its potential effects on inflammation [15], [16] and lipid metabolism [18] that may indirectly alter platelet activities. Compared to the control group, Dp-3-g significantly inhibited platelet aggregation in human and mouse PRP and purified gel-filtered platelets. Dp-3-g also markedly reduced thrombus growth in anticoagulated human and murine blood in perfusion chambers *ex vivo*, as well as decreased platelet deposition, destabilized thrombi, and prolonged the time required for thrombus formation and vessel occlusion in intravital microscopy and carotid artery thrombosis models *in vivo*. We found that Dp-3-g inhibited platelet activation, which broadly affected platelet α- and δ-granule release and cytosol protein secretion. Dp-3-g decreased active integrin αIIbβ3 expression on the platelet surface, and attenuated the binding of Fg to platelets without directly interfering with αIIbβ3-Fg interaction. Dp-3-g also decreased pAMPK, which may in turn decrease αIIbβ3 activation through its inside-out signaling. Thus, inhibition of platelet activation may be the mechanism that explains the anti-thrombotic effects of Dp-3-g observed in the present study.

The effects of some anthocyanin products on platelet aggregation have been studied in human whole blood and PRP after consumption [1], [41], [42]. However, most studies *in vivo* have focused on the effects of food containing anthocyanins, instead of pure anthocyanin compounds or its bioactive derivatives, leading to contradictory conclusions regarding their effects on platelet function. In previous studies, it was demonstrated that querecetin did not significantly affect platelet aggregation [21], [22]. To date, only one investigation assayed the anti-platelet activity of physiologically relevant concentrations of four purified anthocyanins *in vitro* and found that anthocyanins inhibited TRAP-induced platelet aggregation but did not influence platelet reactivity when stimulated by collagen or ADP [20]. In the present study, however, we found that the anthocyanin Dp-3-g inhibited platelet aggregation in both human and mouse PRP stimulated not only by TRAP, but also by ADP and collagen. In addition, although there were several studies examining the effect of anthocyanins on platelet function in whole blood and PRP, there is no report describing the direct effects of these chemicals on platelet function. Using gel-filtered platelets, in which other molecules in the blood plasma are removed, we found that the anthocyanin Dp-3-g directly inhibited platelet aggregation (**Figure 1** **and S1**). Thus, in addition to possible roles in the inhibition of inflammatory cytokines [15], [43] and elevation of high-density lipoprotein [17], [18], which may indirectly affect platelet activities, Dp-3-g can inhibit platelet activation independent of other blood components.

While platelet aggregometry is commonly employed to assess platelet function, it cannot examine platelet aggregation under flowing, pathophysiological conditions. The perfusion chamber is an *ex vivo* model of thrombosis that has a number of important advantages over aggregometry, including the ability to assess thrombus formation on a pathophysiologically relevant substrate and under flow conditions with different shear stresses. A previous study used the Gorog Thrombosis Test to examine the anti-thrombotic effect of whole mulberry fruits under flow conditions [44]; however, it is difficult to elucidate the effect of anthocyanins using whole mulberry fruits. Moreover, thrombus formation cannot be accurately monitored using the Gorog Thrombosis Test. Therefore, we employed the perfusion chamber model and monitored the effects of the purified anthocyanin Dp-3-g on thrombus formation using a confocal intravital microscopy system [27], [31]. We clearly demonstrated that Dp-3-g markedly reduced thrombus growth at both low and high shear rates using both human and murine blood at doses as low as 0.5 µM (**Figure 2** **and S2**). These data suggest that Dp-3-g may inhibit thrombosis at both venous and arterial shear rates.

A role for red wine in supporting cyclic flow reductions (CFRs) in coronary blood flow has been observed using the Folts model of mechanically stenosed coronary arteries and intimal damage [45]; however, the role of anthocyanins in mediating thrombus formation at the site of vascular injury has not been previously studied. Using intravital microscopy, we observed an inhibitory effect of the anthocyanin Dp-3-g on thrombus formation in FeCl_3_-injured mesenteric arterioles and carotid arteries (**Figure 3A and 3B**). Therefore, data from our experiments in *ex vivo* perfusion chambers and the *in vivo* thrombosis models are consistent with our results from *in vitro* platelet aggregation assays. It is notable that the inhibition of thrombus growth in these *ex vivo* and *in vivo* models may not only be due to effects on platelet aggregation, but may also result from the inhibition of platelet adhesion. We found significantly less adherent platelets in the perfusion chambers (**Figure 2** **and S2**), and there was a trend toward decreased platelet deposition at the site of vessel wall injury at the early stages of thrombus formation in the intravital microscopy model (**Figure 3**). This impairment in platelet adhesion may be due to decreased platelet activation since we did not observe that Dp-3-g interfered with GPIb complex-VWF interaction induced by botrocetin and ristocetin (data not shown), which is an important interaction for platelet adhesion at high shear stress [**5**]. Thus, Dp-3-g inhibited platelet activation, adhesion and aggregation, the key events of thrombosis.

Platelet activation plays an important role in inflammation and the initiation of atherosclerosis, as well as thrombus formation in acute manifestations of atherosclerotic diseases [7]. It has been reported that cocoa flavanol and procyanidin supplementation for 28 days significantly decreased P-selectin expression [46], and that cocoa consumption reduced ADP- or epinephrine-stimulated platelet P-selectin and PAC-1 epitope expression and platelet microparticle formation *in vivo* [47], and suppressed unstimulated and stimulated platelet activation in whole blood *ex vivo* [48]. Our data showed that Dp-3-g can not only inhibit ADP-, collagen-, TRAP- and thrombin-stimulated P-selectin, CD63, CD40L, and PAC-1 epitope expression, but also suppresses Fg binding to platelets in both human PRP and gel-filtered purified platelets (**Figure 5** **and** **6**). The active conformations of integrin αIIbβ3, detected by the conformation sensitive monoclonal antibody JON/A and Fg binding, and cell surface P-selectin expression in ADP- and thrombin-activated mouse platelets, were also inhibited by Dp-3-g (**Figure S3**). We found that Dp-3-g did not interfere with Fg binding to immobilized purified αIIbβ3 integrin *in vitro* (data not shown). Thus, we propose that the effects of anthocyanins on platelets may be due to inhibition of platelet activation, which subsequently decreases platelet α- and δ-granule release and cytoplasmic factor secretion, as well as inhibits integrin αIIbβ3 activation and ligand binding.

The mechanism by which Dp-3-g inhibits platelet activation is currently unclear. However, several publications suggested that some polyphenolic compounds can affect platelet signaling pathways. Cinnamtannin B-1, a naturally occurring trimeric A-type proanthocyanidin, shows anti-thrombotic actions through inhibition of Ca^2+^ mobilization and subsequent aggregation in platelets [49]. Delphinidin, a dietary anthocyanidin, inhibits platelet-derived growth factor ligand-receptor (PDGF-PDGFR) signaling [50]. Genistein affects Ca^2+^ mobilization, subsequent platelet degranulation and aggregation. Both quercetin and apigenin have been shown to inhibit kinase activation, which may also be involved in the impairment of platelet responses [51], while quercetin alone has been shown to inhibit collagen-stimulated platelet activation through inhibition of multiple components of the GPVI signaling pathway [52]. Until the present study, however, there have been no studies to address the effects of a pure anthocyanin compound on platelet signaling pathways.

AMPK, a key sensor of cell energy status, has recently been demonstrated to be involved in platelet signaling. Activated platelets can induce pAMPK generation, which may induce phosphorylation of the cytoplasmic tail of integrin αIIbβ3 via an intracellular signalling cascade [40]. This inside-out signaling may lead to conformational changes in the extracellular domains of αIIbβ3 and affect ligand binding, which is required for clot retraction and thrombus stability [36], [40], [53]. Our data clearly demonstrated that Dp-3-g can down-regulate the phosphorylation of AMPK in both human and mouse platelets following stimulation of platelets with the pathophysiological agonist collagen (**Figure 7**), which may attenuate αIIbβ3 inside-out signaling and inhibit platelet aggregation and thrombus growth. Our data, however, cannot exclude the possibility that other platelet signaling pathways may also be inhibited by Dp-3-g. This question, including the possible tissue-specific signaling pathways mediated by anthrocyanins in platelets versus other cells [1], [51], [53], merit further investigation.

Anthocyanins are natural pigments responsible for the blue, purple, red and orange colors of many fruits, vegetables and other plants. Daily consumption of total anthocyanins has been estimated to be between 3 and 215 mg/day [54], [55]. The concentration of anthocyanins in plasma ranged from 0.5–1.0 µM 1.5 hours following consumption [56], [57]. Furthermore, anthocyanins have been reported to show different pharmacological properties at high concentrations, including immunomodulatory actions, anti-inflammatory actions, low-density lipoprotein inhibition, and the reduction of CVDs [58]–[60]. In the present study, the concentrations of the anthocyanins used are within the range between the physiological level and the pharmacological dose (0.5–50 µM) [20]. It is conceivable that the dose to attain the anti-thrombotic effects may be lower in those who have sub-optimal hemostatic factors (e.g. conditions of hemophilia or von Willebrand disease) since we found that Dp-3-g more potently inhibited platelet aggregation of β3-integrin heterozygous mice compared to their wild-type littermate controls (data not shown). Therefore, it is likely that the effects we observed will contribute to the protective and potential pharmacological effects of anthocyanins against thrombosis and CVDs.

Collectively, this study, to the best of our knowledge, is the first to show the effect of the anthocyanin Dp-3-g on platelet function. We found that Dp-3-g significantly inhibits human and murine platelet aggregation, including that with purified platelets, and attenuates thrombus formation at both arterial and venous shear stresses. We determined that Dp-3-g can directly inhibit platelet activation induced by various agonists, which likely explains its inhibitory roles in platelet aggregation and thrombus formation. We further demonstrated that Dp-3-g can attenuate platelet AMPK phosphorylation induced by collagen. Our study suggests that daily consumption of anthocyanins may play an important protective role against CVDs, including decreasing platelet-mediated inflammation and atherosclerosis. The mechanisms of this inhibitory effect on platelet activation, particularly on platelet signaling pathways, merit future studies.

## Supporting Information

Figure S1 **Effects of Dp-3-g on mouse platelet aggregation.** Mouse PRP and gel-filtered platelets were pre-incubated with control buffer (black), 0.5 µM (blue), 5 µM (green) or 50 µM (red) for 40 min at 37°C. Aggregation of mouse PRP and gel-filtered platelets were performed at 37°C with a stir speed of 1000 rpm using an aggregometer. A) Mouse PRP. B) Mouse gel-filtered platelets. Values are mean ± SD, n = 3 per group.
(TIF)

Figure S2 **Effects of Dp-3-g on mouse thrombus formation under flow.** *Ex vivo* thrombus formation was monitored on type-I collagen at 600 s^−1^ or 1800 s^−1^ using mouse whole blood with different concentration of Dp-3-g and control buffer. A) Low shear rate of 600 s^−1^. B) High shear rate of 1800 s^−1^. Values are mean ± SEM, n = 5 per group.* *P*<0.05, ** *P*<0.01 and *** *P*<0.001, as compared to control buffer.
(TIF)

Figure S3 **Effects of Dp-3-g on mouse platelet activation and fibrinogen binding.** Mouse PRP and gel-filtered platelets were incubated with control buffer, 0.5 µM, 5 µM or 50 µM for 40 min at 37°C. Platelet activation markers were analyzed via flow cytometry after stimulation by ADP, collagen, TRAP and thrombin. A) P-selectin expression on platelets in mouse PRP. B) P-selectin expression on mouse gel-filtered platelets. C) Activated integrin αIIbβ3 expression on platelets in mouse PRP. D) Activated integrin αIIbβ3 expression on mouse gel-filtered platelets. E) Platelet-bound fibrinogen in mouse PRP. F) Platelet-bound fibrinogen on mouse gel-filtered platelets. Values are mean ± SEM, n = 3 per group. *P<0.05, ** *P*<0.01 and *** *P*<0.001, as compared to control buffer.
(TIF)
