# Supplementary figures and images for: Plant Food Delphinidin-3-Glucoside Significantly Inhibits Platelet Activation and Thrombosis: Novel Protective Roles against Cardiovascular Diseases
Source: PLoS One. 2012 May 18;7(5):e37323. doi: 10.1371/journal.pone.0037323 (PMC3356278; doi:10.1371/journal.pone.0037323)

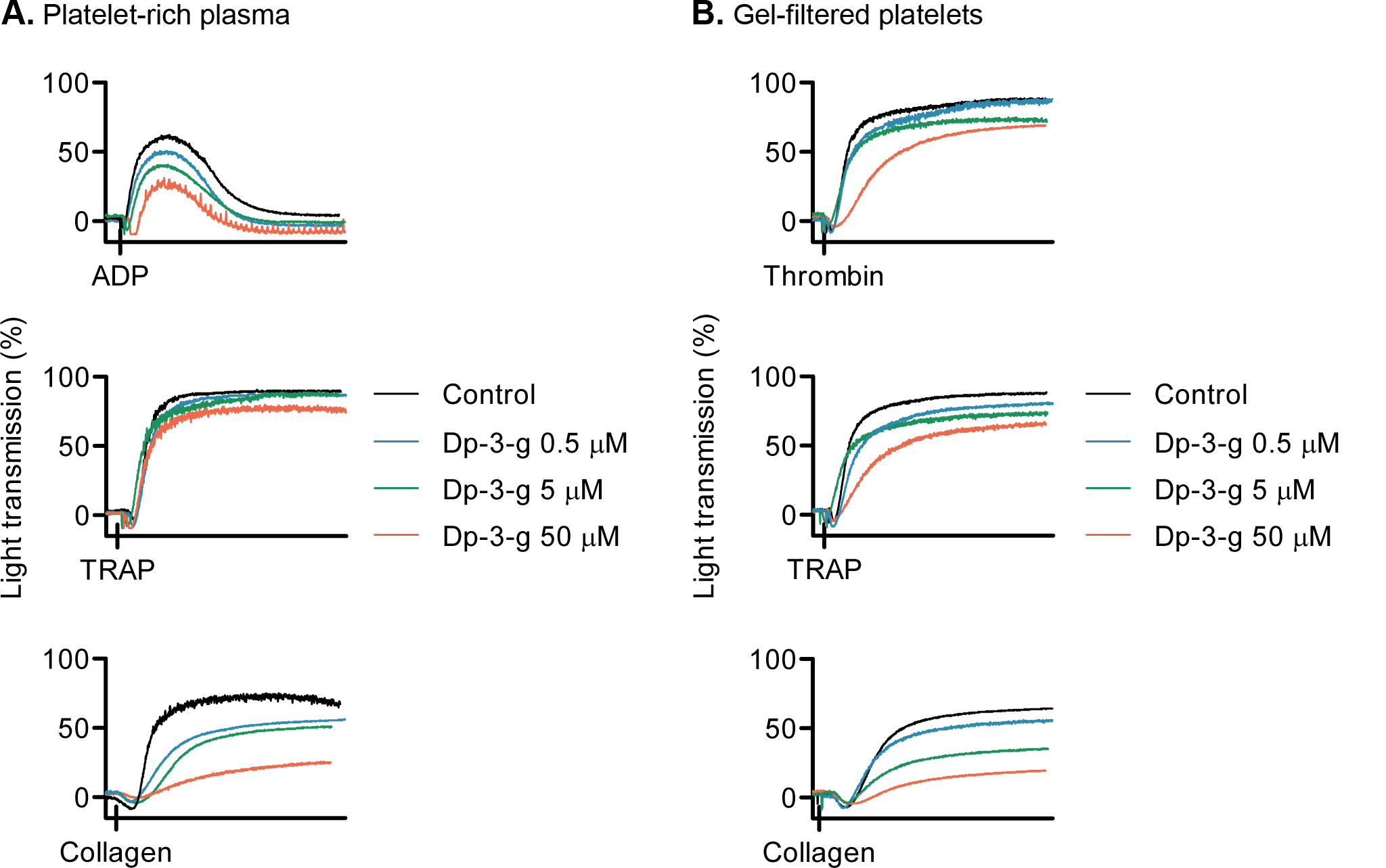

Supplement: Figure S1 — Effects of Dp-3-g on mouse platelet aggregation. Mouse PRP and gel-filtered platelets were pre-incubated with control buffer (black), 0.5 µM (blue), 5 µM (green) or 50 µM (red) for 40 min at 37°C. Aggregation of mouse PRP and gel-filtered platelets were performed at 37°C with a stir speed of 1000 rpm using an aggregometer. A) Mouse PRP. B) Mouse gel-filtered platelets. Values are mean ± SD, n = 3 per group. (TIF) [file pone.0037323.s001.tif]

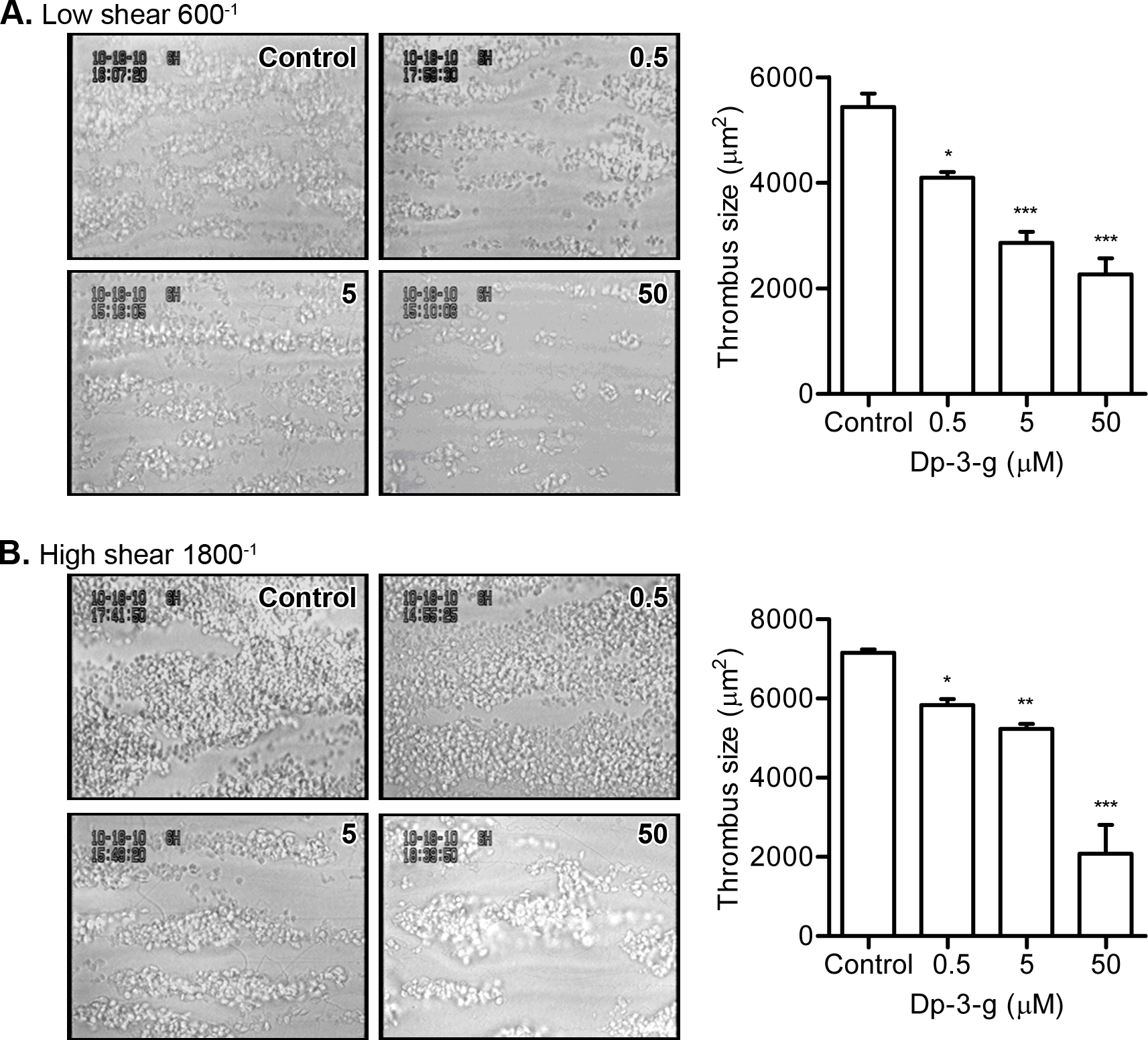

Supplement: Figure S2 — Effects of Dp-3-g on mouse thrombus formation under flow. Ex vivo thrombus formation was monitored on type-I collagen at 600 s−1 or 1800 s−1 using mouse whole blood with different concentration of Dp-3-g and control buffer. A) Low shear rate of 600 s−1. B) High shear rate of 1800 s−1. Values are mean ± SEM, n = 5 per group.* P<0.05, ** P<0.01 and *** P<0.001, as compared to control buffer. (TIF) [file pone.0037323.s002.tif]

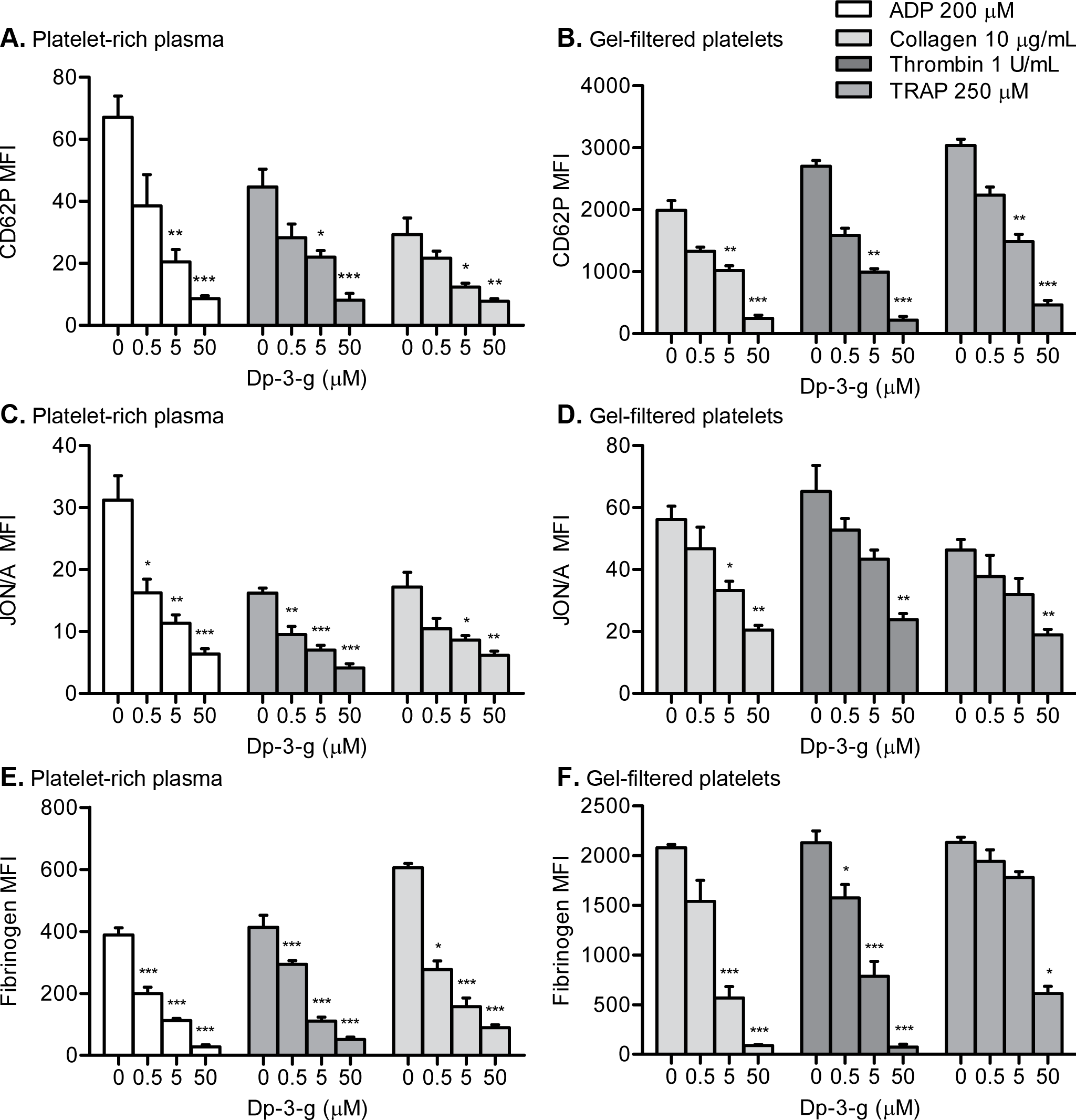

Supplement: Figure S3 — Effects of Dp-3-g on mouse platelet activation and fibrinogen binding. Mouse PRP and gel-filtered platelets were incubated with control buffer, 0.5 µM, 5 µM or 50 µM for 40 min at 37°C. Platelet activation markers were analyzed via flow cytometry after stimulation by ADP, collagen, TRAP and thrombin. A) P-selectin expression on platelets in mouse PRP. B) P-selectin expression on mouse gel-filtered platelets. C) Activated integrin αIIbβ3 expression on platelets in mouse PRP. D) Activated integrin αIIbβ3 expression on mouse gel-filtered platelets. E) Platelet-bound fibrinogen in mouse PRP. F) Platelet-bound fibrinogen on mouse gel-filtered platelets. Values are mean ± SEM, n = 3 per group. *P<0.05, ** P<0.01 and *** P<0.001, as compared to control buffer. (TIF) [file pone.0037323.s003.tif]
